# ‘I can do things that others can’t’: Civic policing as weaponized volunteering in eThekwini, South Africa

**DOI:** 10.1177/00113921221086823

**Published:** 2022-05-14

**Authors:** Tessa Diphoorn, SJ Cooper-Knock

**Affiliations:** Utrecht University, The Netherlands; University of Edinburgh, UK

**Keywords:** Civic policing, South Africa, violence, volunteering, weaponized

## Abstract

In this article, we analyse civic policing in post-apartheid South Africa as a form of ‘weaponized volunteering’. We use ‘weaponized volunteerism’ as a conceptual lens to refer to practices that rest on the potentiality and/or willingness to use physical violence or to harness the physical violence of others under the guise of ‘volunteer work’. By drawing from ethnographic fieldwork conducted by both authors in eThekwini, South Africa, we show that by framing civic policing as weaponized volunteerism, we are able to analyse the violence at the core of policing and underline the varied ways that violence work is harnessed and expanded through civic policing, in the interest of civic and state actors. This, in turn, allows us to explore the continuum between state and civic violence, which is often directed towards similar groups and individuals.

## Introduction

Over the last few decades, there has been a wealth of interdisciplinary scholarship that has questioned the police’s place in the broader policing landscape, pushing us to reappraise who conducts policing, what they do and why. A key concern in this literature, particularly that steered by anthropologists, has been to understand the degree to which the state’s monopoly over violence is accepted or contested on the ground. To that end, much attention has been given to the legality of violence being threatened or deployed by non-state actors and its implications for state sovereignty (e.g. Buur, 2006; Diphoorn 2016; Hansen, 2006; Lar, 2018; Pratten and Sen 2007; Rodgers, 2006; Sieder, 2011). This scholarship has given us incredibly rich insights into statehood and other forms of public authority across the globe (Lund, 2006). Yet the risk of focusing on the violence exercised by non-state actors is that we primarily think of these actors as being engaged in ‘violence work’ when they are exercising force themselves. In the context of civic policing, this means that we risk overlooking putatively non-violent actions that are, in fact, directing and amplifying the violence of the state. In this article, we argue that the concept of ‘weaponized volunteering’ helps us to capture this dimension of civic policing and redress this balance.

The notion of ‘weaponized volunteering’ brings violence back into the centre of our understanding of *all* police work. By applying this concept to civic policing, we speak to all the instances in which citizens mobilize to undertake violence work themselves, or to gain access to the (potential) use of state violence. Thus, rather than questioning the legality of the use of force by citizens within diverse forms of civic policing (see Goldstein, 2012; Kyed, 2009, 2018; Samara, 2010; Vigneswaran 2020) and understanding how this relates to the state police’s use of force (e.g. Bruce, 2002; Hornberger, 2013; Steinberg, 2008), we emphasize the ways in which violence is harnessed and shared more broadly across institutions, regardless of its legality.

We make our claim by drawing from our ethnographic work into civic policing in eThekwini, South Africa, and we focus specifically on initiatives in areas designated ‘white’ suburbs under apartheid.^1^ In this context, we argue, the concept of weaponized volunteering can be particularly useful in helping us to rebalance the broader literature on everyday policing in post-apartheid South Africa. To date, this literature has tended to minimize the direct role that white citizens (and affluent citizens more broadly) play in the ‘violence work’ of policing (Smith 2019: 154). Instead, it has primarily focused on the outsourcing of policing (and, therefore, violence) to private security companies (e.g. Diphoorn 2016; Lemanski, 2006; Marks and Wood, 2007; Kempa and Singh, 2008; Steinberg, 2008; Samara, 2011; Steinberg and Marks, 2014). This is understandable, given the prevalence of private security in affluent areas, such as these. Moreover, this body of work has provided important insights into the practices of private security, and its broader consequences. Valuably, such work also brings the state into view as part of a pluralised policing landscape (Marks and Wood, 2007; Berg, 2010; Berg and Shearing, 2015). And yet, we believe that more attention could be usefully given to the engagement that white South Africans have in the ‘violence work’ of policing (Siegel 2018) through their own policing efforts and their direct relationships to state policing. We are interested in the terrain that the term ‘weaponized volunteerism’ can cover in this direction.

The concept of weaponized volunteerism, however, is also useful beyond the South African context, for two main reasons. First, it underlines the varied ways that violence work is harnessed and expanded through civic policing, in the interest of civic and state actors. Second, weaponized volunteering provides another avenue to explore the continuum between state and civic violence, which is often directed towards similar groups and individuals (Hornberger, 2013). This perspective problematizes the distinction between non-violent and violent forms of civic policing (see also: Benit-Gbaffou, 2008: 94), and aims to gain better insight into the complex relationship between policing, violence and statehood.

The remainder of the article proceeds as follows. First, we clarify how civic policing can be defined as weaponized volunteering and how this allows us to explore the centrality of violence in policing. We thereafter briefly introduce how we researched this topic and our rationale for focusing on formerly white areas in post-apartheid, urban South Africa. In the third and largest section, we explore the empirical foundation for weaponized volunteering and show how (1) citizens become weaponized by the state, (2) how citizens harness the violence work of the police and (3) how violence is enacted by citizens. In our conclusion, we explore the broader applicability of a term that allows us to analytically hold both the violence at the heart of the state project and the violence that spills out or exists beyond its borders.

## Civic policing as weaponized volunteering

In this section, we illustrate why civic policing should be seen as a form of weaponized volunteering. Civic policing refers to policing practices and actions that centre around citizen involvement and are largely instigated by citizens. This includes neighbourhood watches and various types of civilian policing (Crawford and Lister, 2004), as well as forms of state-led community policing, such as community policing forums (CPFs). As an encompassing term, we aim to encapsulate the complexity of policing initiatives that gravitate around citizens.

The definition above may be seem neat, yet in practice, defining civic policing is a messy affair because such initiatives are varied, nebulous, and diffuse across time and space. In particular, civic policing initiatives often blur the line between state and non-state, paid and unpaid (Pratten and Sen 2007). To the degree that people engage with civic policing because they feel unsafe, such operations may also be said to blur the line between recruitment based on consent and compulsion. These definitional struggles are important, but they are not unique to civic policing. Rather, they are a facet of much civic work, as exemplified by discussions over ‘volunteering’.

As the growing, critical literature on volunteering demonstrates (Shachar et al., 2019), what counts as ‘voluntary work’ is a contested terrain. For all its complexity then, we argue that civic policing can be classified as a form of ‘volunteering’. In making this claim, we draw on an established line of work in volunteering studies that explores violence work and volunteering. The roots of the term ‘voluntary’ (*voluntaire*) are military in origin and a strand of literature remains focused on the phenomenon of volunteering for state armies (e.g. Brett, 2003; McMahan, 2013), rebel groups (e.g. Wood, 2003) and transnational armed groups (e.g. Acciai, 2019). This sits alongside literature on police voluntarism that explores the motivations of these volunteers and analyses their role within the policing landscape (see Ayling, 2007; Bullock, 2014; Dobrin, 2017; Löfstrand Hansen and Uhnoo 2020; Millie, 2016, 2019). In the context of South Africa, Kirsch’s (2017) valuable work explores how debates over the parameters of ‘voluntary work’ play out on the streets, when civic policing actors claim to be working for the ‘common good’.

Building on this rich literature, our interest in this section is specifically in the concept of ‘*weaponized* volunteering’ and the conceptual utility that this holds. We argue that to ‘weaponize’ a practice is to enable violence to operate in or through it. The term ‘weaponized’ thus refers to any practice that rests on the potentiality and/or willingness to use physical violence or to harness the physical violence of others. Exploring civic policing as a form of ‘weaponized volunteering’ is, we argue, to focus on the relationship between policing and violence from a fresh direction, which generates valuable analytical insights.

In the rich literature on civic policing that has developed over the last 30 years, one key exploration has centred on the question of violence in relation to sovereignty (e.g. Akarsu, 2020; Bertelsen, 2009; Buur, 2006; Comaroff and Comaroff, 2006; Rodgers, 2006; Ruteere and Pommerolle, 2003; Vigneswaran, 2020). In other words, the degree to which civic actors involved in policing accept the state’s claim to a monopoly over legitimate force. Thus, one key question has been whether or not citizens exercise violence in the pursuit of policing, and if they do, whether they can do so with impunity (e.g. Akarsu, 2020; Goldstein, 2012; Kyed, 2009, 2018; Pratten and Sen, 2007; Sieder, 2011; Super 2017; Yonucu, 2018). In post-apartheid South Africa, the implicit or explicit argument in much of this work is that civic violence undermines the realization of constitutional rights, which were at the heart of South Africa’s transition to democracy (e.g. Oomen, 2004; Posel, 2004). Wresting violence from the hands of the state is seen as a means of undermining the reach of liberal democracy within the country, which occurs either because the state is seen as ineffective or because it is pursuing a vision of security and justice that does not cohere with that which these civic actors hold (e.g. Buur, 2006; Petrus, 2015; Sekhonyane and Louw, 2002; Smith, 2015; Super, 2017). Similar questions have also been asked of collaborative work with the police (e.g. Hornberger, 2013) and private security (e.g. Diphoorn, 2016).

In short, accounts of civic policing have largely been concerned with the exercise of illegal violence: delineating its boundaries; exploring its rationales; and analysing its consequences. The risk in this approach, however, is that civic actors who are not directly exercising violence are seen as non-violent citizens and are therefore often excluded from analytical purview. Through the notion of weaponized volunteering, we explicitly include putatively non-violent forms of civic policing, highlighting their connection to state violence.

Such an argument starts from the premise that violence sits at the core of state policing (Bittner, 1985). As police studies scholars have long highlighted, it is the police’s discretionary capacity to use non-negotiable force that separates them from other state officials (Brodeur, 2007). State police are, in Micol Siegel’s (2018) words, engaged in ‘violence work’ (p. 9). While this does not mean that police consistently use violence in their everyday work, the capacity and potentiality to use violence ‘is the essence of their power’ (Siegel, 2018). From this premise, we argue that to support the police is to support the exercise of violence in the ordering of society (Joseph-Salisbury et al., 2021). When civic policing rests on a collaboration with the state police, as many community policing initiatives do, this stance pushes us to acknowledge that putatively non-violent practices may actually harness the state’s capacity for violence. Recognizing this is important because it shifts how we think about civic action, violence, and democracy. Put simply, using the concept of ‘weaponized volunteering’ reminds us that the contexts in which civic actors *do not feel* as if they need to deploy violence themselves to be secure are as significant as the contexts in which they do. What we ultimately need to understand is whether civic actors who do not exercise violence directly are trying to create non-violent forms of ordering or whether the order they value is defended by state violence (or other forms of violence), which they seek to harness.

For ultimately, while there is undoubtedly widespread impunity for illegal police action (Nyawasha and Mokhahlane, 2017), there is also a great deal of police action that is *not* illegal that could still be seen as undermining the claims to equality and dignity on which substantive democracy supposedly rests (Bonner et al., 2018: 4). In South Africa, as in every country across the globe, the police do not deploy violence evenly. Instead, police work – and the violence on which it rests – ‘serve as a constant reminder of who belongs, and on what terms, in a particular community’ (Cooper-Knock 2020, see also Akala, 2019; Loader and Walker, 2007; Wairuri, 2018). Those who are ‘over policed and under protected’ (Muir in Macpherson, 1999: 312) are constantly reminded that their lives do not count.

Moreover, this uneven distribution of violence is not a sign of the system malfunctioning: it *is* part of the system. This is one of reasons why liberal police reforms that emphasize training, accountability to the law and demographic representation from oppressed groups have tended to fail (Bayley, 2008; Vitale, 2017). Such reforms do not disrupt this logic within the police or the broader criminal justice system (Murakawa, 2014), which remains the management of those ‘on the losing end of economic and political arrangements’ (Vitale, 2017: 32; Neocleous, 2021).^2^

It is important to clarify our intentions in this analysis. Our interest is not to exceptionalize the degree to which civic policing initiatives call upon state violence. Nor is it to suggest that different forms of violence are necessarily equivalent in their form, function, meaning, or consequences. Moreover, while the focus of this article is the violence of policing, it is important not to lose sight of other forms of violence that people face. This is particularly true in countries with comparably high violent crime, like South Africa. Our aim is not to take a normative stance on policing *per se* in this article. It is simply to encourage a focus on the violence in all forms of policing, acknowledging the role that both legal and illegal violence can play in shaping people’s access rights and recognition; life and death. Through the framework of weaponized volunteering, we are able to explore civic violence and its instrumentalization by the state (e.g. Kyed, 2009) as well as the instrumentalization of state violence by civic actors as analytically important practices.

In sum, we argue that ‘weaponized volunteering’ shifts the academic conversation on violence and civic policing in a subtle but important way. Rather than focusing on the wielding of illegal violence, we want to emphasize the significance of violence more broadly. In doing so, we highlight the ambiguous relationships between policing agents, and eschewing any easy distinction between violent and non-violent civic policing (Benit-Gbaffou, 2008:94). Through weaponized volunteerism, we are better able to explore the continuums between state and civic violence, as well as those between legal and illegal violence, which may be directed towards similar groups and individuals (Cooper-Knock, 2020; Cooper-Knock and Super, 2022; Super, 2021).

## Researching civic policing in South Africa

We use the term civic policing to refer to policing practices and actions that centre around citizen involvement and are largely instigated by citizens. In post-apartheid South Africa, this covers a broad range of initiatives, which are often entangled with the state police and private security found in the country (see Diphoorn and Kyed, 2016). The policing landscape in post-apartheid South Africa has been fuelled by a high level of violent crime, which disproportionately impacts lower socio-economic groups, and a broader fear of crime, which captures more diffuse anxieties in post-apartheid South Africa (Marks and Wood 2007, Steinberg 2008).

In both of our research projects, we tried to navigate through this complexity to understand how civic policing fits within the crowded policing landscape. We each conducted ethnographic fieldwork on policing and security in eThekwini, South Africa, between 2007 and 2013. Diphoorn conducted 20 months of ethnographic fieldwork between 2007 and 2010 and focused on the private security industry in Durban and the various ways in which it interacted with other policing entities. By focusing on four different companies operating throughout the city, she was able to observe and analyse diverse policing efforts, such as CPFs and neighbourhood watches, in various parts of the city through conducting participant observation; a range of structured, semi-structured and open interviews; collecting life histories; and analysing secondary data.

Cooper-Knock conducted 10 months of ethnographic fieldwork and interviews between 2009 and 2013 on policing sectors that fell within the police precincts of three different police stations: KwaMashu, Chatsworth and Berea. The latter area, and the life histories of residents in this area, form the basis for this article. Cooper-Knock explored how citizens tackled theft and robbery in each sector, what tactics they used and who they called upon for help. In doing so, she analysed how different policing actors operated in parallel, collaboration and conflict in each of the areas and what this tells us about ideas and practices of statehood, sovereignty and belonging in post-apartheid South Africa. In addition to observing CPF meetings and community patrols, she conducted a total of 170 interviews across the three sectors with local residents, private security workers, and officials. This article thus draws from insight from two different, yet similar research projects that were conducted in the same urban centre within an overlapping time period.

In this article, we will draw from the empirical material that we collected in formerly white suburbs. The spatial legacies of apartheid’s racial segregation in South Africa remain notable (Christopher, 2005; Malala, 2019). Some formerly white suburbs have long been racially diverse and others have shifted in their racial demographic, following ‘white flight’ from areas like Durban’s Central Business District (Firth, n.d.). The areas that we studied, however, remained predominantly white at the time of our research, and civic policing initiatives that we explored were (during our time of research and to our knowledge) predominantly so. We see this focus on white residents in predominantly white areas as a useful narrowing of our analytical lens in this article. In doing so, we are not suggesting that the policing work and preferences of white South Africans are homogenous or completely distinct (Clarno and Murray, 2013: 223). We are simply stating that they deserve greater analystical attention. Given that our research is based in urban areas, we will not be addressing violence by white citizens in rural areas, where the histories and contemporary realities of policing are comparable but distinct (Bolt, 2016; Higginson and Strobel, 2003). Thus, our analysis intentionally gives a partial insight into specific areas. Nonetheless, we believe that it provides a valuable corrective to the broader literature on civic policing in South Africa and, in doing so, illustrates the utility of ‘weaponized volunteering’ as a term.

## Weaponized volunteering in South Africa

As mentioned above, our empirical material in this section will draw from the accounts of white residents in predominantly white, urban residential areas. We see this as a useful means of demonstrating the utility of weaponized volunteering. The existing literature on civic policing in post-apartheid South Africa, we argue, has predominantly focused on areas that were assigned as ‘black’, ‘coloured’ and ‘Indian’ areas under apartheid (e.g. Buur, 2006; Hansen, 2006; Jensen, 2008; Petrus, 2015; Tshehla, 2002). In the literature on predominantly white residential areas, there is a tendency to focus on private security and the *outsourcing* of violence. In this article we want to show the crucial role that white citizens play in the ‘violence work’ of policing beyond their engagement with private security. This element of violence work, we feel, is often overlooked in the research on policing in urban, post-apartheid South Africa.^3^ As we will demonstrate, it includes both the ways in which they harness state violence and how they instigate violence themselves, in ways that can be harnessed by the state.

Through this focus, we are not implying that weaponized volunteerism is absent in other neighbourhoods. On the contrary, weaponized volunteering is a concept that can be used to explore civic policing across the country. Yet it is arguably most useful in uncovering the violence of relatively privilege citizens who, due to the structural position they hold, are more likely to have recourse to state policing or private security services.^4^ The advantage of the term, however, is that it does not necessarily suggest that those who can access violence will always choose to do so. In this section, we demonstrate the conceptual utility of weaponized volunteering in practice by outlining that ‘weaponized volunteering’ includes civic actors who are utilized violently by the state, who rely on state violence and those who deploy violence directly.

### ‘Take back your streets!’: state utilisation of civic violence

White vigilantism was a resilient feature of colonial and apartheid South Africa, entangled with state projects of coercion, domination and rule (e.g. Murray, 1989; Evans, 2013). Often, this violence underwrote or extended the state’s violent white supremacy (Keegan, 1987; Higginson and Strobel, 2003). The living legacies of apartheid and the violence on which it rested are crucial to comprehend. In contemporary South Africa, for example, militarised white masculinities are refracted through these histories of violence and statehood (Langa et al., 2020). At the same time, many of the white South Africans that we interviewed depicted themselves and their neighbours as fundamentally law-abiding, distancing themselves from any association with violence (Cooper-Knock 2016; see also: Smith 2019: 154; Bolt 2016:914).

In this section, we begin to explore ‘weaponised volunteerism’ in formerly white suburbs, starting with the state’s utilisation of civic violence. We argue that state officials in post-apartheid South Africa have encouraged civic policing initiatives, which include coercion and force. The active role of the state police in weaponizing citizens, we argue, must be analysed within a wider neoliberal framework, where we see the specifics of South Africa policing become enmeshed with an increasing focus on responsibilization and securitization (Kirsch 2017; Marks et al., 2009).

Securitization refers to the ways in which socio-economic issues not directly related to security, such as homelessness, are framed primarily as threat to the safety of others (Samara, 2011). Studies across the globe have demonstrated a tendency to securitize issues that could otherwise be seen as socio-economic issues arising out of structural injustices (Cortes-Nieto and Ansari, 2017; Vitale, 2017). Scholars like Vitale (2017) argue that community policing increases the number of issues that are defined as ‘police issues’: in other words, issues that can be legitimately answered by the reinforcing of order based on coercive force.

If securitization legitimizes weaponized interventions, then responsibilization shapes our ideas of who should be responsible for those interventions. Responsibilization refers to the broader ways that citizens are held responsible for accomplishing things that would previously have been seen as beyond their purview (Rose, 1996). In security studies, this has typically been associated with the state insisting that the citizens take greater responsibility for their own security (see Garland, 1996) and act as ‘responsible citizens’ (Johnston, 1992). As we will see below, some citizens have resisted this, mobilizing in order to try and pull the state into their lives. Nonetheless, we witnessed and heard of numerous instances in which the police sought to task citizens with responsibility with sourcing their own weaponized solutions, through either the hiring of private security or direct action. This presented two key benefits for state officials: First, it reduced the pressures upon an arguably over-stretched and under-resourced police service. Second, it diluted the responsibility that police officers held for the exercise of violence and coercion. In our work, police officials, private security workers and citizens each spoke about the ways in which the police utilized the violence of non-state actors who were less likely, in their eyes, to be held to account for their violence (Diphoorn, 2016; Cooper-Knock, 2016). This echoes the findings of studies of police work elsewhere (Buur, 2006; Kyed, 2018).

Much of the work of this securitization and responsibilization in South Africa happens through community policing forums (CPFs). CPFs were implemented during the early stages of the transition to act as the institutional lynchpin to increase state legitimacy and build relationships of trust between citizens and the state police (Gordon, 2001; Shaw, 2002).^5^ By 1995, CPFs were compulsory in police stations across the country (Cawthra, 1997), with CPFs also sometimes existing at a sector level (the sub-areas that exist within each station’s precinct). Initially positioned as more agonistic spaces aimed at enhancing police accountability (Dixon, 2004; Gordon, 2001), the policy vision for CPFs quickly shifted, as citizens were placed into more of a supporting role (Gordon, 2001: 133).^6^ Meanwhile, during the 2000s, police tactics became increasingly combative as successive police commissioners promised brutality against criminals in the ‘war’ on crime (Steinberg 2014). By 2010, then National Police Commissioner Bheki Cele asserted that criminals would have to ‘pray to their god devil’ for protection from the police (*The Sunday Tribune*, 28 November 2010). Arguably, since the Marikana Massacre in 2012 – which saw 34 striking miners killed by the police–this discourse has been moderated. Nonetheless, the basic orientation remains, as the appointment of Bheki Cele as Minister of Police in 2018 made clear. These shifts have had contradictory impacts upon CPFs. On the one hand, forums have been side-lined in the policing landscape, and conceptualised support structures to the police. On the other hand, some citizens have become emboldened by the violence of the state, which mirrors their own stance on policing and punishment (Hornberger, 2013). Since their invention, CPFs have morphed into diverse policing structures: some have a conciliatory tone towards the state, while others are more combative; some have periods of active membership, while others garner skeletal numbers; some are primarily social gatherings, while others are active in fighting crime. Such dynamics also change over time. In the sector that Cooper-Knock studied, for example, there had previously been a more active neighbourhood watch. By the time of her research, however, this had largely demobilised and civic policing tended to emerge in the midst of a suspected crime (Cooper-Knock 2016).

The emboldening of citizens – which we see here as a process of weaponizing – clearly emerged during CPF meetings that we attended where citizens were encouraged to ‘take back your streets’, to create neighbourhood watches and to be ‘vigilant’. This was made explicit during a CPF meeting that Diphoorn attended in February 2009 (see Diphoorn, 2016: 169). When the crime patterns of the area were deliberated, a specific road was identified as a ‘problem’ due to the large number of recent break-ins. The police officer coordinating the meeting then issued the following warning:

If no one in that road does anything, they will get punished. They will get robbed, or even worse. And then if that happens, they will come crying to the police and blame us for not being there. But this is not only our responsibility: you are responsible for your road; you cannot expect us to do everything.

This statement shows how citizens were encouraged to assume some responsibility for their own safety. Furthermore, while accompanying police officers on their patrols, Diphoorn frequently heard how they blamed citizens for certain crime incidents. Police officers often held the sentiment that citizens relied too much on them and that they needed to take particular matters into their own hands, which included subscribing to a private security company or setting up a neighbourhood watch.

This, of course, was a conflicted process: in some cases, police officers sought to mobilize civic actors within strict parameters and would virulently oppose those who shifted from being the ‘eyes and ears’ of the police to ‘taking the law into their own hands’ (Cooper-Knock, 2014). In other cases, police officials may play lip service to this principle but, in practice, were willing to allow residents much greater latitude for direct violence (Cooper-Knock, 2016; Diphoorn 2016). In her fieldwork, Diphoorn witnessed how police official overlooked the use of intimidation and physical violence by residents to ‘secure their neighborhoods’. Either way, we can see community policing as a means through which citizens become weaponized. In the case of more affluent residents, weaponising occurs both when they are encouraged to engage directly in police work and when they are encouraged to fund private policing in their areas, although civic policing remains the focus of this article. We contend that it is important to acknowledge this dynamic because it highlights that weaponized volunteering is used by the state, as well a way in which the state is used.

### ‘Get the police back’: civic utilisation of state violence

For citizens, CPF activities are also spaces in which they can elicit a response from the police by building relationships with them and essentially harnessing violence from the police. Residents who are involved with CPFs, as attendees of meetings, financial contributors, or active patrollers, often see their role as trying to (re)build the relationship between themselves, their broader neighbourhood and the police. Often, membership within these forums surged in the wake of a personal crime or a crime that was considered notable within the neighbourhood. Responses such as ‘I want a safer community’ or ‘we need to come together to fight crime’ were common. More specifically, most people attended because they sought to elicit a response from the police that they felt was lacking. Our concern in this section is not looking at the *success* of these measures, but their intention and what this can tell us about their relationship to violence. At the broadest level, people joined the CPF because they wanted the police to be more active and, specifically, to be more active in policing their areas and addressing their concerns.

In Berea, for example, a whole host of activities were recounted to Cooper-Knock during her research. From braais to prayer meetings, most of these activities were intended to deepen the relationships between people and ‘their’ police (see Hornberger, 2004). Take, for example, the braai that was organized by a regular CPF attendee, Dean, who also played a central role in the local neighbourhood watch. As Dean explained,

The primary aim for this braai is to get the police back. You give them the food, you give them the braai and you give them the opportunity and they will be there. . . we need to establish the relationship again with the police. A firm one, where they recognize our faces, etcetera. (Cooper-Knock 2014: 189)

This use of food as the basis of a social relationship with the police has been well documented beyond the CPFs (Vigneswaran and Hornberger, 2009). What we see here is an attempt to build a sense of mutual connection and obligation so that people could call on ‘their police’ when needed (Hornberger, 2004).

In the example above, Dean was seeking to cultivate relationships between the police station and his local policing sector, but this pattern replicated itself at an individual level: People often used the CPFs to try and solicit the numbers of police, who could be called when needed. Arguably, the CPF played a particularly important role in harnessing the police for those in affluent areas, who were less likely to have friends, family or neighbours in the police and therefore have the kinds of bonds that facilitated contact calling.

As mentioned, our aim here is not to uncover whether these attempts to bolster the police institutionally and make them more responsive were successful. In practice, this was a flawed process that was punctuated by frustrations and disappointments on both sides. While many CPF members in the forums managed to secure the personal number of police officers in their local area, the degree to which they could secure a response varied. In the Berea CPF, one white, middle-aged, police official was, in the words of a CPF regular, ‘willing to dish out his number’ to residents (Cooper-Knock 2014: 189). They welcomed the sense of personal connection that this brought. As Samantha reflected, ‘I have the telephone number of Johannes and yeah, you know, I feel like I have a personal link to the Police’ (Ibid). However, this apppeared to be more of a gesture of goodwill on Johannes’ part than a commitment to become residents’ personal policing service. Like Naomi, several residents reported that ‘the odd time that I have phoned him and it is just a tape-recorded message so it does not help really, you know?’ (Ibid). As a result, some became highly disillusioned. One despondent former CPF chair branded her local forum a ‘melting pot of nothingness’ (Ibid). These irritations were not universal. For some individuals, the personal connections with police officers seemed to be more consistent, as they were for Dean. What interests us, however, is that residents *sought* to create a more responsive police service. If our interest is state sovereignty, the legal use of police connections is not particularly notable. In this article, however, we are interested in ‘violence work’. In this context, calls to the police are notable because they summon state actors whose authority is directly underwritten by the threat of force.

Of equal interest to us here are the forms of policing that people demanded. In reports shared at CPF meetings and messages shared on SMS networks that both authors studied, residents were seeking action against ‘suspicious’ people. As numerous studies have shown, this was a label predominantly assigned to young, black men who were seen in the area, particularly those who appeared to be poor (Benit-Gbaffou, 2008; Samara, 2010; Clarno and Murray, 2013; Diphoorn, 2017). It was often this profiling (along the lines of race, class, and gender) that prompted calls to the state police and private securit (Diphoorn, 2017).^7^ Although police officials sometimes voiced frustrations at these call-outs, the same logic was often echoed in the state’s own patrol work (Steinberg, 2008; Samara, 2011). In making this argument, we are not saying that all calls to the police were unwarranted and discriminatory and we do not aim to exceptionalise the behaviour of CPF members. All citizens, we would argue, remain complicit in the state violence that they utilise and that which they do not actively resist (Alves and Costa Vargas, 2017). Nonetheless, the fact that CPF members actively mobilize themselves to create a more responsive police service, harnessing a service that is based on the threat of force, makes it fitting to use the label ‘weaponized volunteerism’.

The analysis above demonstrates the importance of moving beyond a fixation on questions of legality in our analysis of violence. In this section, we want to show the importance of complicity and support for the violence work of state policing. According to this definition, the only instance in which CPF members would not be considered weaponized volunteers would be if they joined a CPF explicitly to oppose any use of force within policing. We have found no evidence of any CPF members adopting this role. While a few (particularly in the early days of the CPFs) may have joined to redirect, monitor or limit the polices’ use of force, they were not essentially contesting the use of force by the police *per se* (see also: Marks and Wood, 2007:150).

### ‘I can do things that others can’t’: Civic violence in action

In addition to harnessing violence, the policing activities linked to CPFs are often sites where state violence is expanded, both explicitly and implicitly. This predominantly occurs through CPF members using or threatening to use violence in their policing efforts, often with the implicit support of police officers. In line with what has been documented by other scholars working on civic policing elsewhere (see Akarsu, 2020; Di Nunzio, 2014; Kyed, 2009, 2018), we observed and heard how various members of civic policing initiatives used violence or threatened to use violence in determining access to certain areas, intimidating and threatening ‘suspicious’ others, and instilling a certain moral order more broadly (Diphoorn, 2016; Cooper-Knock 2016).

Diphoorn observed this during a night patrol in April 2019 with a neighbourhood watch operating in one of Durban’s middle-to-upper class neighbourhoods. This neighbourhood watch was not formally tied to the local CPF, yet they were closely intertwined in various ways. First, the regular patrollers were active members of the CPF, which was described as a functional and ‘successful’ one. In fact, Billy, who we will meet, was the chairperson of a particular sector within the CPF and was regarded by other members as the ‘driving force’ of the entire CPF. It was a CPF that met regularly, was well-attended by residents from the neighbourhood and had set up a range of schemes to harness trust between the community and the police, such as fundraising events. On top of that, the CPF, like many others, had been able to draw from an existing neighbourhood watch that had existed for many years prior.

During this night patrol, Diphoorn accompanied a group of armed, white men in their privately owned cars to patrol specific areas in the neighbourhood, based on recent crime incidents. The patrol was rather uneventful and the majority of the time was allocated to checking out certain ‘hot spots’ of crime, such as a park known for drug dealing, and stopping at points where something ‘suspicious’ was happening, such as an intoxicated man stumbling down a road. The tone of the patrol changed when the patrollers spotted a few black men sitting in a combi van that was not currently operating as a taxi.^8^ Billy and another male patroller approached the van’s passengers and after a rather cordial conversation, the patrollers eventually became very verbally hostile and firmly asked them to ‘Get out of here’ because ‘there was nothing to find here’. Their instructions were met and the van left the neighbourhood.

At the end of the shift, the patrollers explained that questioning such individuals was a common occurrence. One of the patrollers stated that their main aim was to ‘keep crime out’, that is, out of their neighbourhood, and this involved ‘keeping certain people out’. Billy also explained that tomorrow he would contact the local police officers and give them an update of this patrol, ‘to keep them informed’, thereby reaffirming the working relationship between the neighbourhood watch and the local police station.

This patrol is an example of the ways that residents mobilize themselves to police their own neighbourhood and how this occurs within complex policing constellations. This neighbourhood watch, for example, was largely run by a police reservist and is embedded within the formal structures of the local CPF, that is, an institutional interface between the state police and residents. Furthermore, the neighbourhood watch worked closely with a private security company that had two armed response officers permanently stationed in this area.

With this patrol, we want to underline two key issues. The first crucial point that is evident from the patrol described above is the ways in which the patrollers intimidated the people in the van who were ‘suspects’ in their eyes, using coercion and intimidation to drive them from the area. From other interviews that Diphoorn conducted with various patrollers from this neighbourhood watch, it would seem that the use of physical force to intimidate, apprehend and arrest ‘suspects’ was certainly not rare. In addition to this patrol, both authors observed throughout their fieldwork how various civic policing initiatives would attempt to intimidate, manhandle or assault those walking through their neighbourhoods who were deemed to be ‘suspicious’. Highlighting such violence is crucial, as is understanding the tacit support that it receives both from members of the broader neighbourhoods these groups purport to protect and from the CPFs they may visit. While not dismissing the contestation that such violence could provoke, we want to highlight the support and complicity of many residents and state officials in such practices. When attending some of the CPF meetings, for example, Diphoorn observed how their patrols and ‘policing ways’ were supported and encouraged. This is a way, we argue, in which CPFs expand the violence inherent to police work. Moreover, as mentioned above, this civic violence amplifies – rather than disrupts – broader patterns of state violence, in that it is exercised predominantly at those assumed to be poor, young, black males entering the area. In this context, we see that rights-based democracies may not universally constrain violence, they may also legitimize (or render invisible) particular forms of violence (Alves and Costa Vargas, 2017; Perry, 2013).

Finally, we want to emphasize the overlapping forms of volunteerism that exist in this example. In addition to leading this civic policing initiative, Billy was a police reservist – a volunteer for the state police. Reservists, of whom the vast majority are male, are currently defined as ‘a member of the community, who volunteers their services to perform policing functions or activities for the South African Police Service without being remunerated for such service’ (SAPS website). Police reservists were active in every CPF structure Diphoorn encountered in her research, highlighting the influential role that they have played in shaping the South African policing landscape, both historically and in the current era (Bezuidenhout, 2017). Formally emerging in the 1960s (Forster-Towne, 2013), reservists have long been seen as ‘force multipliers’ for the police, constituting over one-seventh of the SAPS police service in some provinces (Forster-Towne, 2013). Reservist volunteering is seen as bolstering the employment prospects of lower socio-economic groups (Forster-Towne, 2013; Hirtenfelder, 2016). For many of the more affluent, white reservists that Diphoorn spoke to, however, becoming a reservist was a way of getting involved with civic policing and ‘do[ing] things that others can’t’. In another part of Durban, for example, Mike was a reservist who patrolled his community in his free-time, managed a company that was involved in ‘private-security related work’ and had a high-ranking position on the provincial CPF structure. This stands in tension with the National Instruction of 2002, which forbade reservists from being actively involved in CPFs or working in the private security industry. In understanding how violence is harnessed across institutions, we need to incorporate the crucial role that such volunteers play in mobilizing other citizens and how this occurs through habitual movements across and beyond the borders of state and non-state.

By employing the notion of weaponization, we thus aim to emphasize the support for violence that exists within policing initiatives operating under the beacon of civic policing and thus highlight people’s broader support or opposition to forms of ordering underwritten by violence that function across state and society. Weaponization, therefore, provides a lens for understanding how policing efforts occurring within civic policing structures are ways in which state violence is harnessed and expanded.^9^

## Concluding remarks

In this article, we have proposed to use the framework of weaponized volunteerism to analyse various forms of civic policing in South Africa. Drawing from our ethnographic research projects in eThekwini, South Africa, we have tried to show how weaponized volunteerism provides a different perspective on civic policing. The term ‘weaponized’ centralizes the role of violence in policing and demonstrates how the use (or threat) of violence, is distributed. Civic policing structures, we argue, may provide opportunities for citizens to harness or expand state violence and thereby reproduce certain repertoires of violence. This is particularly problematic for those who are ‘over policed and under protected’ such as the young black men who are regularly harassed and intimidated.

With this term, our aim has also been to problematize any violence continuum that posits state and civic violence at opposing ends. Rather than solely showing that civic policing efforts can also be violent, as has been demonstrated by others, we have tried to portray how support for violence does not only occur through the use physical violence but also through supporting and accessing services that operate with the threat of force. We thus take this support of violence in a broader sense and include those who do not use violence, but mobilize themselves as part of civic policing structures because they actively seek to support, strengthen and harness this weaponized arm of the state. Weaponized volunteerism allows us to encapsulate the ways in which a wide group of actors reproduce orders underwritten by force that systematically marginalise certain individuals and groups. It does so, however, without necessarily creating an equivalence between differe.

For us, the notion of weaponized volunteerism has been helpful in understanding much of what we observed in our research projects, but we also believe that it can serve to understand community policing efforts elsewhere. As highlighted by scholars such as Bertelsen (2009), Goldstein (2012) and Rodgers (2006), civic policing operates within complex policing constellations, and the (potential) use of violence is often a key defining feature. In this article, we concentrate on analysing weaponized practices.

The open question that remains, however, is whether all projects of civic policing – even those that seek to create alternative orders to those of the state – are necessarily weaponized projects. Might there be ‘regimes oriented around projects of care’ (Seigel, 2020) where ordering is not grounded in violence? Do all acts of ordering ultimately rest on the threat of non-negotiable force? Or is this only true in contexts where the forms of order being pursued are fundamentally exclusionary and unjust (e.g. Samara, 2010)? Such questions are at the centre of many debates around police abolitionism in the recent years. While we do not have sufficient space to highlight them in this article, such questions demonstrate the salient and timely discussions that can be sparked by discussions of weaponized volunteering. In this article, by analysing community policing in formerly white suburbs in South Africa as forms of weaponized volunteering, we aim to correct a long-running imbalance in the literature on civic policing and prompt further discussions on the complex dynamics between policing, violence and statehood.

## Notes

1. All racial categories are social constructs that carry political weight. In South Africa, racial categories such a those used here have also been deeply implicated in systems of white supremacy. We use them here acknowledging their problems and limitations but also their continued use. The terms we have chosen here were emic terms used by our participants.

2. Such discussions are tied to a broader debate over the degree to which constitutionalism can be a tool for fundamentally restructuring society in South Africa or whether it does little to disrupt the injustices and inequalities of apartheid (Madlingozi, 2017)

3. Discussions of white vigilantism are far more established in accounts of colonial and apartheid South Africa, including Bradford (1987); Murray (1989); Higginson and Strobel (2003); Peté and Devenish (2005); Higginson (2014); Super (2017: 522). Evans (2013) also tackles how the state’s violence could be harnessed and weaponized by white citizens. In the context of post-apartheid South Africa, these discussions are more explore with the exception of Smith (2019), Sekhonyane and Louw (2002), and Bolt (2016), who explores the prolonged presence of white vigilantism and white South Africans’ support for the vigilantism of others.

4. Despite the transformative changes that occurred during the formal transition from apartheid in 1994, we consider the white South Africans with whom we researched to be structurally privileged due to the economic legacies of apartheid and the ways that institutional racism continues to function in South Africa (e.g. Anciano, 2020). That said, we do not underestimate the varied experiences of white South Africans more broadly and the importance of other intersecting structural identities.

5. See Gordon (2001) and Pelser (1999) for more detailed information about how community policing forums (CPFs) operate and their limitations.

6. Despite the fact that CPFs are state-initiated spaces (Ruteere, 2017), we use the term ‘civic policing’ to cover the initiatives that emerge from such forums. We do so in recognition of the fact that even where a policing initiative emerges from a state-initiated forum, it is dependent upon civic volunteerism and its operations are not under comprehensive state control. Moreover, most initiatives that are tied to CPFs do not simply emerge at the state’ behest. Historically, CPFs entered populated policing landscapes and often became embroidered within pre-existing policing structures, such as neighbourhood watches, street committees and private security schemes. Since then, CPFs have served as a hub for civic initiatives; at other times, they co-exist, but the demarcation lines between state and civic actors are flexible and porous (Buur, 2006; Fourchard, 2011; Kirsch, 2010; Kyed, 2018).

7. In the context of the US it is specifically this form of unwarranted, discriminatory reporting that Takei (2018) has conceptualised as the ‘weaponising’ of the state by civic actors. Here, we are using ‘weaponized’ to speak to violence more broadly.

8. In South Africa, ‘taxis’ refer to 16-seater Kombi mini buses. These are the vehicles that form the backbone of South Africa’s transport system. Privately run by different taxi associations with their own routes across the city, taxis are an inexpensive and ubiquitous way to get around. These vehicles may also be owned and used in private hire.

9. What are colloquially known as the ‘July Riots’ in South Africa occurred after the authorship of this paper. However, a recent research trip by Diphoorn suggested that many of the civic policing initiatives emerging in this period to exclude ‘looters’ and ‘outsiders’ were linked to CPF and neighbourhood watch structures. Future research could valuably unpack the relation here between violence, statehood, and civic action.
